# Single-Molecule Optical Biosensing: Recent Advances and Future Challenges

**DOI:** 10.1021/acsphyschemau.2c00061

**Published:** 2023-01-12

**Authors:** Swayandipta Dey, Mathias Dolci, Peter Zijlstra

**Affiliations:** † Eindhoven University of Technology, Department of Applied Physics, Eindhoven 5600 MB, The Netherlands; ‡ Institute for Complex Molecular Systems, Eindhoven, 5600 MB, The Netherlands; § Eindhoven Hendrik Casimir Institute, Eindhoven, 5600 MB, The Netherlands

**Keywords:** Single-molecule sensing, fluorescence, nanoparticles, binding kinetics, continuous monitoring, multimodal sensors

## Abstract

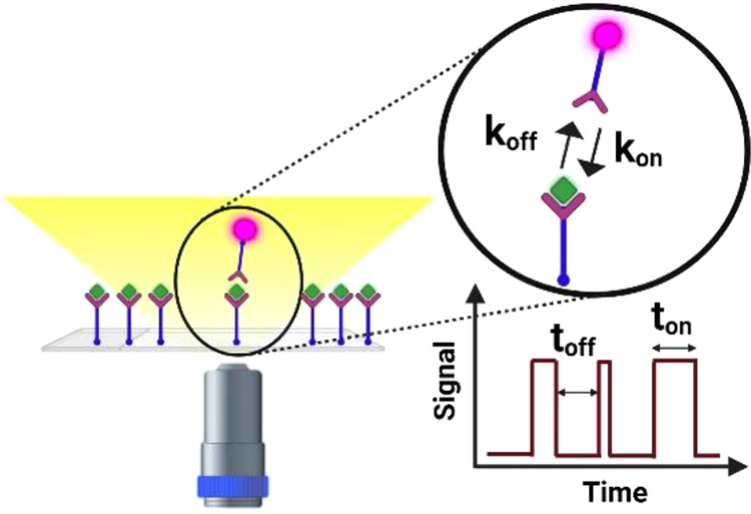

In recent years, the sensitivity and specificity of optical sensors has improved tremendously due to improvements in biochemical functionalization protocols and optical detection systems. As a result, single-molecule sensitivity has been reported in a range of biosensing assay formats. In this Perspective, we summarize optical sensors that achieve single-molecule sensitivity in direct label-free assays, sandwich assays, and competitive assays. We describe the advantages and disadvantages of single-molecule assays and summarize future challenges in the field including their optical miniaturization and integration, multimodal sensing capabilities, accessible time scales, and compatibility with real-life matrices such as biological fluids. We conclude by highlighting the possible application areas of optical single-molecule sensors that include not only healthcare but also the monitoring of the environment and industrial processes.

## Introduction

Biomolecular sensors have become indispensable in the past few decades due to their central role in diagnostics. They typically contain a biological component (e.g., a capture probe or enzyme) that reacts with the analyte of interest and a physical component (e.g., an optical or electrical system) that transduces the reaction to a detectable signal. The majority of biosensing assays are now run in centralized facilities (e.g., hospital laboratories) where polymerase chain reaction (PCR) and enzyme linked immunosorbent assays (ELISA) are the workhorse technologies. Only in rare cases have biosensors been developed that are small and simple enough to be used at the doctor’s office or even at home. The most prominent examples of the latter are pregnancy tests and COVID rapid antigen tests that use a lateral flow assay in combination with gold nanoparticles.^1^ These tests yield a qualitative diagnosis (i.e., positive or negative) without reporting the actual concentration of the analyte that may aid in establishing, e.g., the progression or severity of a condition. PCR and ELISA on the other hand are quantitative approaches and yield the concentration of a certain biomarker at a certain point in time.

Beyond such single-time point measurements, many conditions benefit from a continuous readout because it enables the monitoring of a health condition over time.^2^ Current sensors that continuously monitor a patient often use a wearable device to report physical parameters like temperature, heart rate, and blood oxygenation. Although these are useful markers to establish the health of a patient, they do not contain information on the underlying causes of abnormal readings which are encoded in the concentration profiles of biomolecules. Continuous monitoring sensors for a wide range of biomarkers are therefore considered the next landmark in point-of-care diagnostics and personalized healthcare.

The most successful biomolecular sensor in use today is the continuous glucose monitoring sensor. It provides quantitative measures of glucose concentration over time.^3^ Recently, continuous monitoring sensors have also been developed for cortisol and lactate.^4,5^ The main reason that these sensors are successful is their electrochemical readout with specific enzymes in combination with the high concentration of analytes. However, this sensing concept is difficult to generalize to other analytes, particularly those that are lowly concentrated (picomolar to nanomolar). This has sparked the need for a more general detection mechanism that is suitable for submicromolar concentrations.

In response, affinity-based assays have been developed that capture analyte using receptors that are immobilized on a sensor surface. The binding of the analyte is then transduced to a detectable signal in a label-free manner (e.g., by detecting the shift of a resonance in response to local refractive index changes) or a label-assisted manner (e.g., by detecting the presence of a secondary scattering nanoparticle or a fluorophore). A large body of literature exists on so-called ensemble-averaged affinity-based biosensors^6^ where the signal from a large number of biomolecules is integrated to yield a signal that can be read out by, e.g., mobile-phone-based devices.^7^ Beyond ensemble-averaged sensing, the progress in optical detection technologies has sparked the development of affinity-based optical sensors with single-molecule sensitivity.

Note that, in terms of sensitivity, such single-molecule sensors are in most cases not competitive with ensemble-averaged approaches. The latter reach highly sensitive (fM limit of detection) and highly specific (<1% percent false positive results) diagnostic tests by averaging the signal over a large number of analyte molecules and long times. Single-molecule sensitivity however does have other advantages over ensemble-averaged sensing, particularly in affinity-based assays that reveal the molecular association and dissociation events in real time:

(1) Single-molecule sensors that reveal the association and dissociation of molecules provide digital signals, enabling the direct counting of molecules and/or interactions. Counting is insensitive to the slow drifts (due to, e.g., temperature) that typically interfere with the analogue signals of ensemble-averaged sensors.
(2) It enables the detection of molecular interactions even if the fractional occupancy of receptors is low. At low receptor occupancy, an ensemble-averaged sensor typically does not provide a signal, whereas single-molecule sensors still provide a molecular count.
(3) It enables the analysis of the characteristics of each single-molecule detection event (e.g., the signal amplitude or the bound-state lifetime). This gives access to heterogeneity in the sample and enables the distinction between different populations of biomolecules caused by, e.g., specific and nonspecific interactions.

Several excellent review articles have been published that highlight these advantages of single-molecule resolution in affinity-based bioassays.^8−12^ In this Perspective, we briefly recap highlights from the field in the past years and describe future directions that will enable the progression from single-point diagnostic tests to multimodal platforms for continuous monitoring of single biomolecules. We first introduce the assay formats in the form of direct (label-free) assays, sandwich assays, and competitive assays. We then outline the most important challenges in the coming years focusing on compatibility with complex matrices, continuous monitoring functionality, miniaturization of the device, and the inclusion of multimodal detection technologies. These research directions all contribute to achieving the single-molecule sensing devices for continuous and multimodal monitoring of biomarkers with applications in industry, the environment, and healthcare.

## Current Status of Single-Molecule Optical Sensing

**Figure 1:**
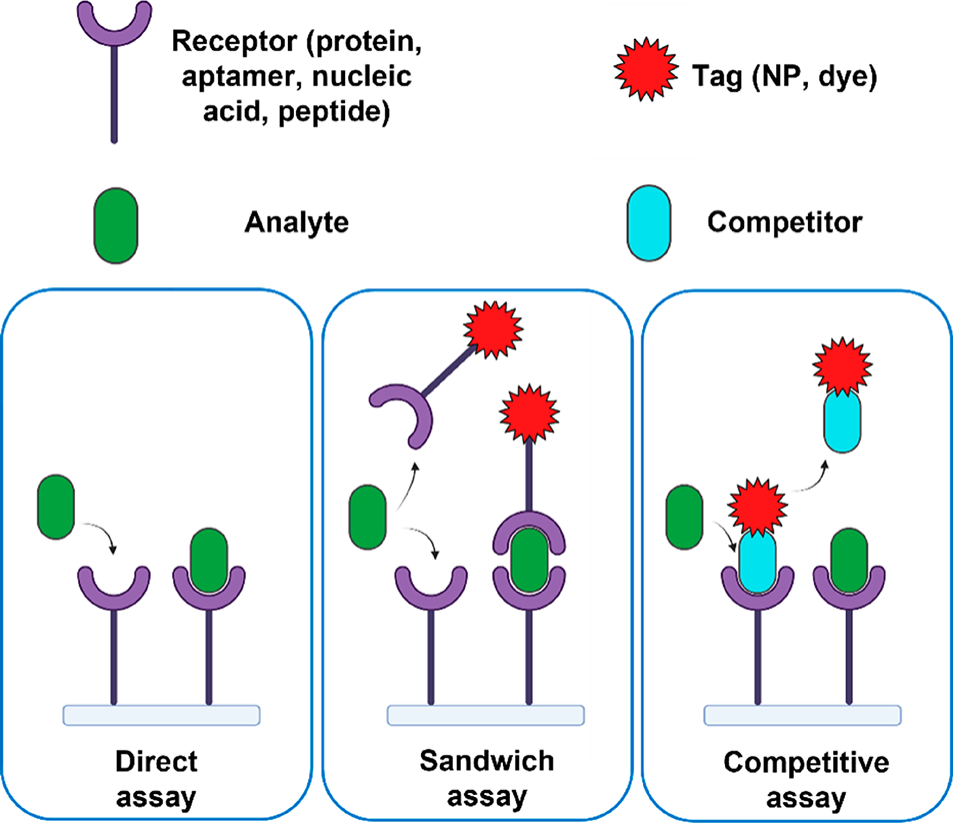
Illustration of three assays used in affinity-based single-molecule sensors. In a direct assay, the signal is generated by the analyte itself. In a sandwich assay, a tag or detection probe is used to provide a signal once an analyte is bound. In a competitive assay, a labeled competitor is detected whose interaction with the receptors is prevented in the presence of an analyte.

We start by describing the optical assays that have been reported in the literature in the past years. They can be broadly categorized as direct assays, sandwich assays, and competitive assays (Figure 1). In a direct assay, the signal is generated by the analyte itself. If the analyte by itself does not generate a sufficient signal, a sandwich assay can be used wherein a tag (e.g., a fluorophore or a particle) is used to provide a signal. This requires two complementary capture probes that bind to different regions on the analyte, which is often not possible when the analyte is a small molecule. In that case, a competitive assay can be used where a labeled competitor provides a signal. In the presence of an analyte, the number of binding sites for the competitor decreases, resulting in an inverse relationship between the number of detected events and the analyte concentration. Below, we highlight examples of single-molecule implementations of these assays.

### Direct Assays

Direct assays are conceptually the simplest sensing architecture through which molecular binding events between analytes and receptors can be directly converted into detectable and quantifiable signals. Over the past few decades, there has been an enormous research effort aiming to develop novel label-free detection methods for direct detection and screening of a wide range of chemical and biological analytes.^13^ Because the direct assay does not require any external tags (like dyes, quantum dots) for signal amplification, the assay is simple and potentially suitable for high-throughput acquisition. In addition, the kinetics of the assay is dictated by a simple one-step binding process providing direct access to kinetic parameters.^14^ Over the past few decades, technological advancement in the areas of optical imaging and nanofabrication have enabled single-molecule assays based on nanoplasmonics, nanophotonics, and scattering-based detection methodologies.^10,15,16^

Commercial devices for label-free detection rely on the capture of analyte on a surface that supports plasmonic or photonic modes.^17^ Analyte binding to the surface results in a change in local refractive index inducing a shift of an optical mode which forms the basis of the readout in most of these evanescent-field based sensors. Although this technology has proven to be a robust analytical tool for quantification of biomolecular interactions and affinities, it is not suitable for detecting single molecules due to its extended surface area over which the signal is averaged.

An alternative and logical approach to overcoming these limitations is by implementing plasmonic nanoparticles (PNPs) that exhibit a far smaller surface area and therefore a larger signal per molecule. PNPs exhibit localized surface plasmon resonances (LSPR) due to collective oscillation of conducting electrons that can be easily tuned by modulating the particle’s size, shape, composition, and local dielectric environment. An added advantage is that the resonance frequency of noble metal PNPs (Au, Ag) occurs at visible–NIR wavelengths (400–1000 nm) and is therefore compatible with most standard optical microscopes. Specifically, the electric field associated with particle plasmons acts as a transducer that converts the change in local refractive index into LSPR frequency shifts. The shift of the resonance scales with the spatial overlap between the biomolecule and the plasmonic mode volume.^18^

**Figure 2:**
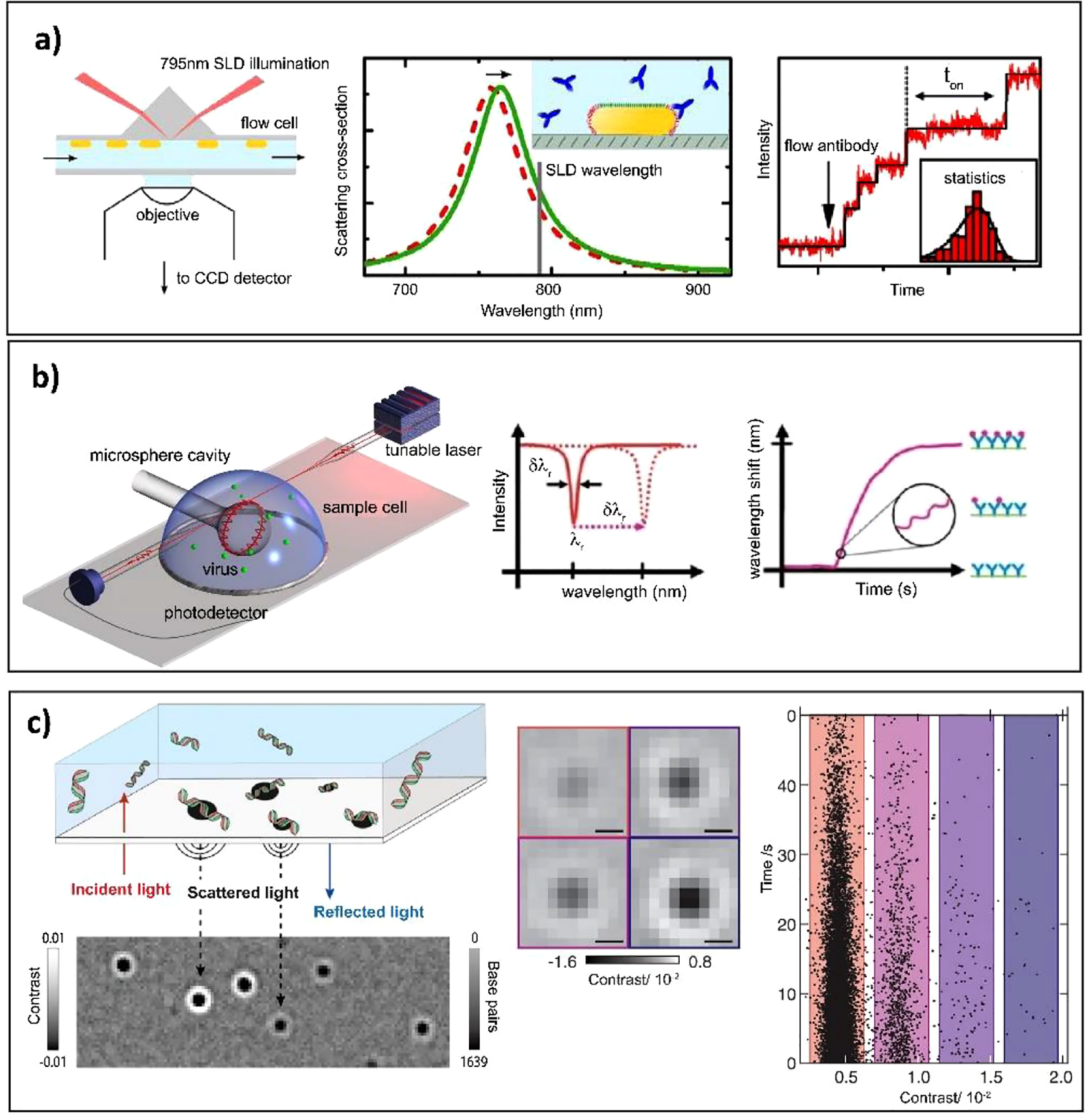
Examples of single-molecule direct assays. (a) Left: Schematic of the optical setup for monitoring stochastic protein interactions using plasmon sensing. Middle: Illustration of detection principle; gold nanorods are functionalized with receptors (depicted in red), whereas the sides are blocked with tetra ethylene glycol (depicted in green). The binding of individual antibodies results in a red shift of the plasmon resonance. Right: Time trace of the normalized scattered intensity of a single gold nanorod. Stepwise changes in the signal indicate stochastic binding of single antibodies. The distribution of waiting times between events is used to determine the antibody concentration. Reproduced with permission from (23). Copyright 2015 American Chemical Society. (b) Left: Experimental design of a Whispering Gallery Mode (WGM) based sensing platform showing detection of single virus particles. Middle: The resonance is identified at a specific wavelength from a dip in the transmission spectrum acquired with a tunable laser. A resonance shift associated with molecular binding; Δλ_r_ is indicated by the dashed arrow. Bottom panel: Binding of analyte is identified from a shift Δλ_r_ of resonance wavelength. Reproduced with permission from (28). Copyright 2008 Proceedings of the National Academy of Sciences. (c) Left: Concept of interferometric scattering mass spectrometry (iSCAMS) and working principle of label-free DNA detection employing iSCAMS. Individual DNA molecules diffusing in solution bind to an appropriately charged glass surface. Middle: Binding events cause changes to the reflectivity of the interface, visualized by a contrast-enhanced interferometric scattering microscope through the interference between scattered and reflected light. Right: Statistics of the image contrast provide a single-molecule readout of molecular mass. Adapted with permission from ref (33). Copyright 2020 Oxford University Press. Adapted with permission from (36). Copyright 2018 American Association for the Advancement of Science.

Plasmon shifts can be easily measured with designated far-field optics operating in different modes in the form of dark-field microscopy (DFM), total internal reflection (TIR) illumination, or even through a photothermal effect.^19^ The first two modes probe scattering cross sections, whereas the latter probes the absorption cross-section of the particle. Additionally, combining far-field optics with PNPs is particularly advantageous since the probe volume (which is determined by the extent of locally enhanced electric field for PNPs) can be more than 10^5^ times smaller than the diffraction limit of light (zeptoliter volumes), thus enabling detection of single molecules even at micromolar concentrations.^20,21^ Zijlstra et al. for the first time reported on the possibility of detecting nonlabeled single molecules in real-time by monitoring the LSPR of a biofunctionalized gold nanorod (AuNR) through photothermal or scattering microscopy (Figure 2a).^22−24^ The sensors consisted of a small AuNR coated with biotin receptors wherein the binding of a single protein molecule resulted in a detectable longitudinal LSPR shift. Around the same time, Ament et al. also reported on utilizing single gold NPs as signal amplifiers to monitor the structural evolution of a single unlabeled fibronectin protein by continuously probing dynamic binding events on a millisecond time scale.^25^ Whispering gallery mode (WGM) based dielectric resonators and optical microcavities are another class of refractive index sensors that has been implemented in biological and chemical sensing.^26^ These sensors use a similar concept as PNPs, wherein molecular binding is detected by shifts in resonant frequency due to changes in the local refractive index near the sensor.

A WGM resonator typically exhibits a low internal loss, resulting in a weakly confined near-field (i.e., a large mode volume) but a significantly increased Q factor (up to 10^6^) compared to their plasmonic counterparts. Such narrow resonance line widths enable measurement of smaller spectral shifts, although there is a trade-off between the weak field confinement and the high Q factor. One of the first works on resonant optical microcavities for label-free single-molecule detection was by Vollmer et al, describing the direct detection of single protein molecules.^27^ Later on, the same research group also reported a label-free, real-time optical detection of Influenza-A virus particles using a WGM resonator, wherein the binding of single virions was observed from discrete changes in the resonance frequency shifts of WGMs excited in the microsphere cavity (Figure 2b).^28^ More recent implementations take advantage of hybrid photonic–plasmonic structures to combine the strong field confinement of plasmonic structures with the high Q factors of photonic structures. Liang et al. and Baaske et al. used such a hybrid sensor to study dynamic DNA–protein interactions with millisecond temporal resolution.^29,30^

Nanoplasmonic and nanophotonic structures possess a limited sensing volume because they can only detect changes in the local refractive index within their mode volume. Interferometric techniques do not require such nanostructures for light confinement, but rather detect the interference signal between a reference beam (often the reflection from the sample’s glass–water interface) and the light that is scattered by a molecule (Figure 2c). The interferometry is crucial here because the scattering signal itself scales with the square of object volume and is very small for single molecules.^31−33^ The interferometric signal scales linearly with object volume and can be detected with careful subtraction of background signals. A systematic development of this interference base started in the early 2000s as a general effort to explore label-free options for studying single molecules that do not suffer from photobleaching or blinking of fluorescent tags. Kukura et al. used this technique for the detection and tracking of single viral particles.^34^ Using such interferometric scattering microscopy (iSCAT), Andrecka et al. performed single particle tracking experiments and investigated the structural dynamics of Myosin 5a motor protein at nanometer spatial and millisecond temporal precision.^35^ Young et al. used this method for mass quantification of proteins with 2% mass accuracy, up to 19-kilodalton resolution, and 1-kilodalton precision.^36^ Very recently, plasmonic sensors were interrogated with iSCAT to probe plasmon shifts of a single gold nanoparticle, enabling the probing of single hemoglobin proteins with nanosecond temporal resolution.^37^ iSCAT has been rapidly advancing, transitioning from single-particle tracking studies in low scattering media to 3D tracking in more complex microenvironments such as cellular membranes.^38^

### Sandwich Assays

Although direct assays are the simplest to implement, they exhibit limited sensitivity, particularly for smaller analyte molecules (<50 kDa), that result in low signals. An alternative is to use a sandwich assay which relies on the binding of a tag that provides the signal. This is a generic approach that works for analytes of any size as long as they have two independent binding sites for recognition of the tag and the capture probe. The detection probe contains a tag that can be of various types, but fluorophores or scattering nanoparticles are the most often used. Molecular detection then involves several steps: (i) a receptor immobilized on the surface of the sensor, (ii) the recognition of the analyte molecule, and (iii) binding of a detection probe to the analyte which generates the signal to be measured (Figure 1). Here, the capture and detection probe have to be complementary; i.e., both can bind to the analyte simultaneously and independently. Sandwich assays are particularly suitable for the detection of single molecules and have been widely used owing to the strong signal generated by each detection event.^8^ The requirement to use a labeled detection probe is therefore quickly outweighed by the efficiency of the method. Most single-molecule approaches are based on the capture of the analyte on the surface of the sensor using a capture probe with a high affinity. This results in the analyte being bound for the whole duration of the experiment, which is then detected by subsequent incubation with the labeled detection probe.

**Figure 3:**
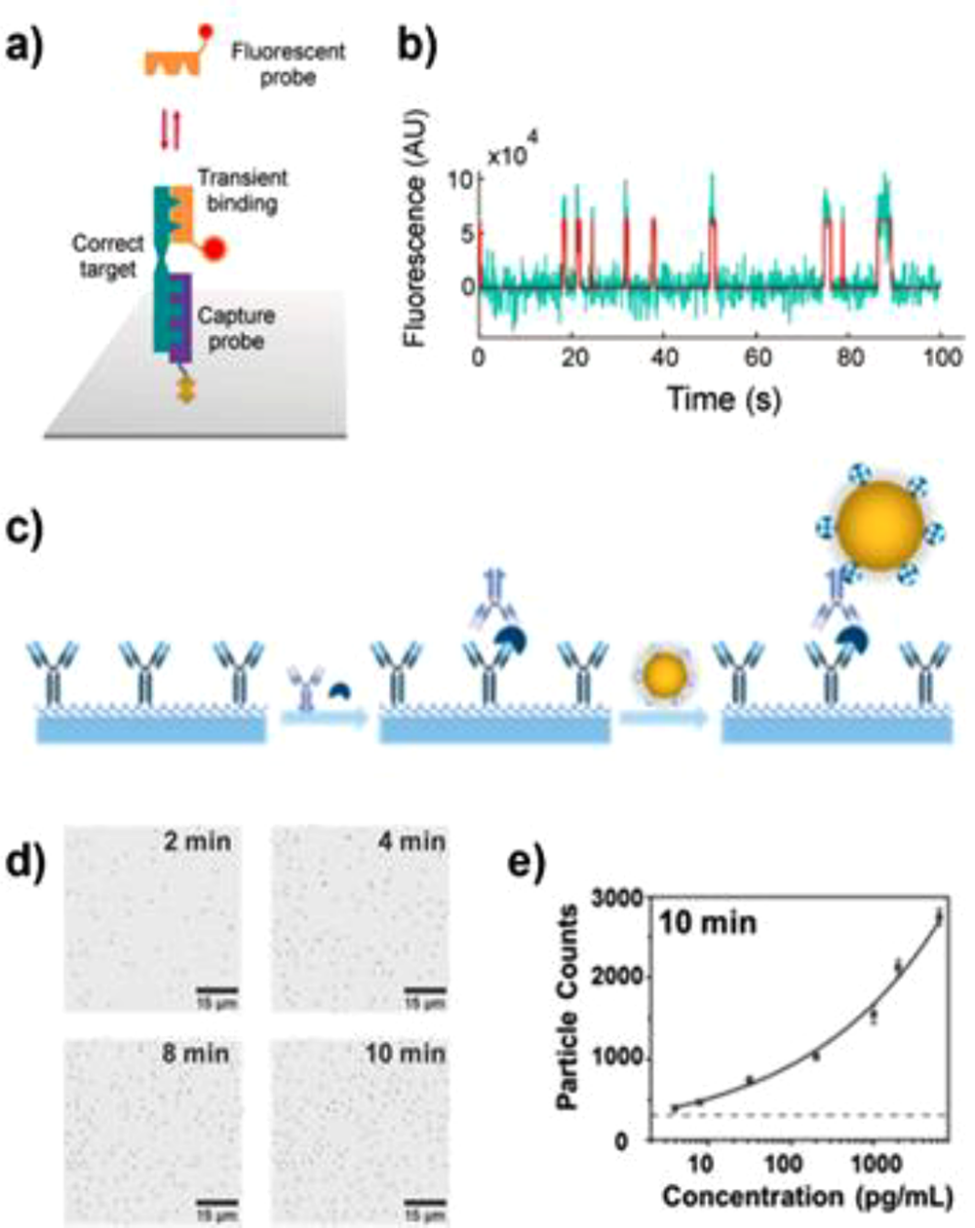
(a) Schematic representation of single molecule recognition through equilibrium Poisson sampling (SiMREPS). (b) Representative intensity versus time traces in the absence and presence of adenosine (50 pM). Reproduced with permission from (39).  Copyright 2020 American Chemical Society. (c) Illustration of one-step process for forming the human PCT antibody–antigen–antibody sandwich complexes. (d) Bright-field images (part of entire view) over time for digital counting. (e) Standard curve of PCT detection at 10 min. The error bars are the standard deviation of triplicate tests, and the dashed line represents the level of blank. Reproduced with permission from (47).  Copyright 2020 American Chemical Society.

The team of Walter takes advantage of this approach by using a fluorescent dye as a tag in the so-called single molecule recognition through equilibrium Poisson sampling (SiMREPS) technique.^39^ In this method, the detection probe has a low affinity for the analyte, resulting in transient binding events (Figure 3a). The use of low affinity detection probes (with dissociation rates in the range *k*_off_ = 0.1–10 s^–1^) results in the repeated association and dissociation of the detection probe on the same analyte molecule, providing multiple detections per analyte. The recorded intensity timetrace (Figure 3b) displays the intensity bursts which correspond to the interaction of detection probe with a single analyte molecule. SiMREPS has enabled the detection of different types of analytes such as nucleic acids,^40−42^ proteins,^43^ and small molecules,^44^ rivalling the detection limit of ELISA.

While having a high signal-to-noise ratio, fluorophores suffer from photobleaching that may affect the detected kinetics of association and dissociation and may lead to a progressive and eventually permanent loss of the detection probe. Nanoparticles, although larger and potentially subject to steric effects, overcome this shortcoming by providing a highly stable scattering signal that does not photobleach. Moreover, they offer a variety of mechanisms to exert a force or torque on the nanoparticle by incorporating magnetic materials or electrical charge, thereby providing dual functionality in terms of optical detection and actuation.^45^

The team of Wang uses gold nanoparticles as detection probe and counts the number of particles bound to an analyte molecule using bright field microscopy. The method relies on the capture of analytes by antibodies that are immobilized on the surface of a coverslip, and subsequent binding of detection antibodies in a sandwich assay. The number of detection antibodies is then counted by introducing streptavidin-coated gold nanoparticle to complete the sandwich assay (Figure 3c). By digitally counting the number of particles in the field of view, the determination of the concentration of cardiac troponin I and procalcitonin could be performed (Figure 3d and e).^46,47^ Moreover, the use of TIR configuration enables the precise tracking of the nanoparticle position, giving insight in the confined diffusion of every bound particle.^47^

The above implementations use tags that bind to the analyte by free diffusion from the bulk solution to the sensor surface. One drawback of this approach is that the mass transport to the sensor surface limits the rate at which any analyte can be detected. Recently, hybrid gold–iron oxide nanoparticles have been implemented to overcome this limitation. Magnetic actuation was used to pull the particles toward the sensor surface, resulting in an increased rate of particle binding compared to passive diffusion.^48^ In addition, to obtain a dynamic signal the authors used the same external force to increase the dissociation rate of the detection probe. This approach provides a dynamic assay similar to SiMRePS with the added advantage that the binding and unbinding kinetics can be tuned by the application of magnetic fields. The actively tuned digital event count enables the detection of microRNA and amyloid-beta proteins with a low limit of detection. Recently, the team of Cunningham proposed a similar approach using magneto-plasmonic particles actuated by a magnetic field at the surface of a photonic crystal to quicken the transport of the particles toward the sensor surface.^49^

Additionally the association process can be accelerated by confining the detection probes near the sensor surface. Several studies achieve this by tethering the detection probe to the sensor surface using a long DNA sequence. Analyte binding events are then detected by analyte-mediated binding of the detection probe to the sensor surface^50^ or by an analyte-mediated conformational change in the tether (see next section).^51^ Apart from fluorophores and nanoparticles, several other labels are used in sandwich assays including quantum dots and up-conversion nanoparticles. The reader is invited to read recent reviews on these single molecule detection systems.^45^

### Competitive Assays

Most of the analytes discussed above exhibit multiple independent recognition domains and are therefore compatible with the sandwich format. However, to detect a smaller analyte with a single recognition domain, this format is no longer suitable. The competitive assay overcomes this limitation as it is based on the presence of a competing detection probe that binds to the same receptor as the analyte. In the absence of analyte, the detection probe directly (and dynamically) interacts with the receptor providing a sequence of detection events. In the presence of the analyte molecule, the frequency or duration of the detection events is reduced because the analyte competes for the same receptors (Figure 1). Competitive assays are widely used in ensemble-averaged sensors to detect proteins, nucleic acids, and other types of analytes^52^ but only rarely used for single-molecule biosensors.

Single-molecule fluorescence sensors have been developed using the competitive assay format for the detection of, e.g., ssDNA.^53^ Herein, a sensor surface was functionalized with receptor DNA and subsequently incubated with fluorescently labeled detection probes that directly bind to the capture DNA. This results in dynamic binding events whose frequency depends on the number of available capture probes. In the presence of the analyte, the receptors are partly saturated due to analyte binding, resulting in a reduction of the number of detected fluorescence events for increasing analyte concentrations.

**Figure 4:**
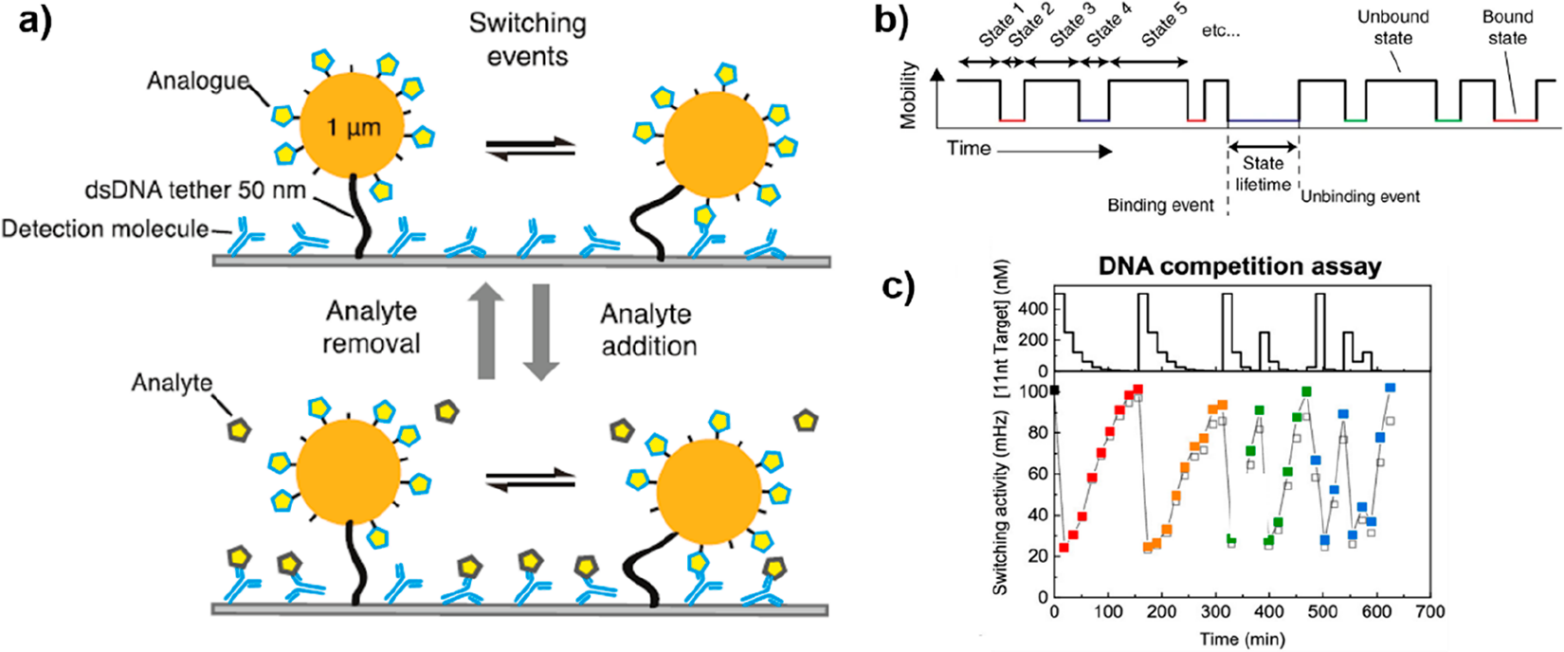
Design of a digital single-particle sensor. (a) Schematic drawing of continuous molecule monitoring with a digital single-particle switch (the molecules are not to scale). The sensing functionality is embedded in the digital switching behavior of the particle. The particle dynamically switches between bound and unbound states because of transient binding between the detection molecule and analogues. Reproduced with permission from (54). Copyright 2020 American Chemical Society. (b) The mobility of the particles is analyzed as a function of time, and the binding/unbinding events are digitally detected for hundreds of particles in parallel. The time between two consecutive events corresponds to the lifetime of the enclosed state. Reprinted with permission from ref (51). Copyright 2018 Nature Portfolio. (c) An example of switching activity measured over time for an ssDNA analyte. The top panel shows the concentration–time profiles, and the bottom panel shows the measured switching activity. The switching activity shows an inverted response (high analyte concentration gives low switching activity), as expected for a competitive assay. Red and orange data points represent equal decreasing concentration series; green and blue data points represent sequences of alternating concentration values. Lines are guides for the eyes. Reproduced with permission from ref (55). Copyright 2020 American Chemical Society.

Another approach toward a competitive assay is to use particles tethered to the sensor surface via double-stranded DNA and monitor their mobility by optical microscopy.^51^ Interactions between the particle and a receptor molecule on the sensor surface result in a confined Brownian motion of the particle. The frequency of switching events between high- and low-mobility states is then used as a readout for the analyte concentration.^54,55^ The use of analyte analogues that are conjugated to particles induces a specific switching activity that is reduced when the analyte is introduced (Figure 4a and b). Recently, the concept has been generalized by using free particles in solution which simplifies the sensor preparation and storage.^56^ The reversibility of the single-molecule interactions here implies that increases as well as decreases in analyte concentration can be measured, resulting in a biosensor that is suitable for continuous monitoring of analyte concentrations for small molecules such as creatinine and cortisol.^54,56^

## Commercial Implementations

The first commercial devices based on the described assay formats have already appeared in past years. Currently, these devices mostly find application in research and development where the sensor provides single-molecule characterization of biomolecular properties. In comparison to ensemble-averaged sensors, single-molecule sensitivity has the advantage that it provides distributions of molecular properties rather than simply the average. This notion is used in, e.g., next-generation sequencers of Pacific Biosciences that use a single-molecule fluorescence readout for nucleic acid sequencing.^21,57^ Single-molecule fluorescence sensing is used to sequence parts of the target DNA strands, and subsequent stitching of different single-molecule reads can be used to obtain the full-length sequence. Additionally, single-molecule protein detection based on interferometric scattering microscopy has recently been commercialized by Refeyn for single-protein mass spectroscopy in heterogeneous samples.^36,58^

These devices operate in clean buffered conditions and are therefore particularly suited for biophysical characterization of biomolecules. In diagnostics applications, single-molecule sensitivity provides a digital readout of the number of detected biomarkers, but the complex matrix (e.g., blood) poses additional challenges in the form of nonspecific interactions. These can be circumvented by, e.g., separation of the analyte from the complex matrix by capturing the analyte on a flat substrate (as is done in the SiMREPS assays commercialized by aLight Sciences^59,60^) or a magnetic bead (as is done by Merck’s Single-Molecule Counter^61^). Following washing steps, single-molecule fluorescence can be used in a clean buffered solution to count the number of captured analytes as is done in, e.g., the SimREPS sandwich assays described above.

Alternatively, single molecules can be directly detected in complex matrices by not using fluorophores but nanoparticles as very bright optical labels. One commercial implementation by Scanogen uses analyte-induced tethering of micron-sized beads by long DNA tethers that are formed in the presence of an analyte.^50,62^ All above diagnostic platforms provide a single measurement point at a time, even though applications would profit from a continuous stream of data points to enable tracking of biomolecular concentrations. Helia Biomonitoring develops continuous monitoring biosensors based on tethered particles that undergo changes in their Brownian motion pattern to detect single-molecule binding events.^51,63^ The use of low-affinity single-molecule interactions provides reversibility to the sensor and enables the tracking of concentration fluctuations in filtered blood plasma.

## Challenges and Opportunities

Future developments of such single-molecule sensors, either for sensors for R&D or for healthcare applications, may enable continuous monitoring of biomonitoring directly in the unfiltered biological fluid. In combination with further miniaturization, this may constitute the next generation of optical affinity-based wearable biosensors. Below, we describe the main challenges in the field that need to be tackled to achieve this ambitious goal.

### Biosensing Across Timescales

**Figure 5:**
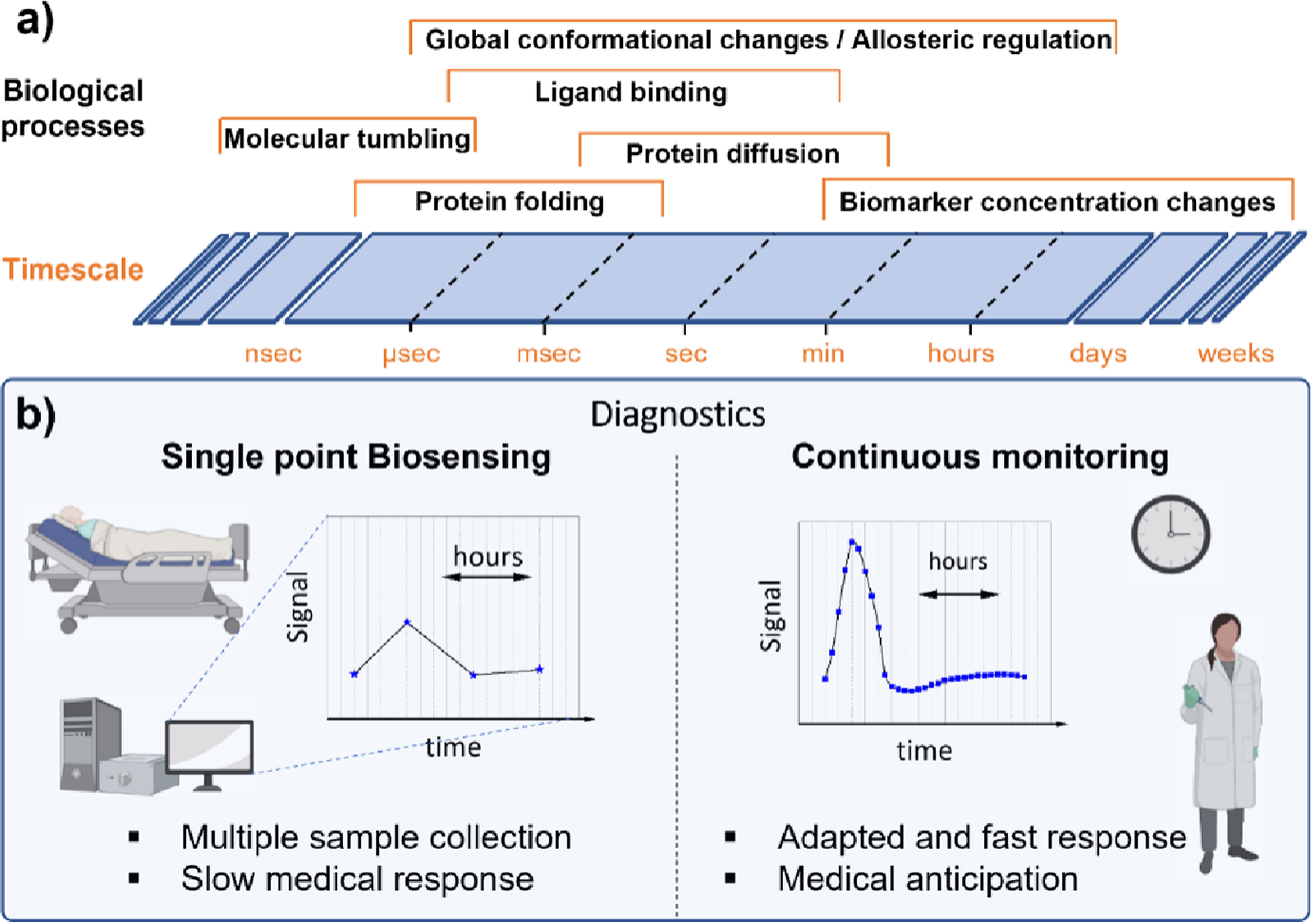
(a) Characteristic time scales for various biomolecular processes. (b) Comparison of single point biosensing and continuous monitoring in terms of diagnostics.

In the field of biosensors, enabling detection across the large range of time scales relevant in biomolecular detection will be a key challenge. On the one hand, single-molecule biosensors show promise for the study of biomolecular processes but have largely focused on the association and dissociation processes that occur on millisecond to second time scales. As shown in Figure 5a a wide range of biomolecular phenomena however occurs on much shorter time scales (submillisecond for, e.g., protein folding dynamics^64^), whereas monitoring the fate of biomarkers (e.g., to follow the progression of a disease like sepsis^65^) requires measurement over a long period of time up to days. Current research and applications of biosensors however largely focus on time scales of milliseconds to minutes due to limitations in the signal intensity (restricting the lower limit) and stability (restricting the upper limit). Development of biosensors that can access shorter or longer time scales is therefore a central challenge to increase the applicability of single-molecule optical biosensors.

For fast processes the signal amplitude provided by single molecule events is often relatively low limiting the integration time of the sensor to milliseconds. For example, the brightness of a single fluorophore is typically limited to 10^4^ to 10^5^ photons/s, prohibiting real-time measurements on time scales of microseconds or shorter. Therefore, it is necessary to be technically capable of operating with high-speed measuring systems but also to be able to provide a sufficient signal for the detector at these time scales. In the case of fluorescence detection, the use of plasmon-enhanced fluorescence provides an effective mechanism to drastically increase the signal produced by a fluorophore.^66^ Gold nanoparticles of different shapes have been reported to enhance fluorescence signals up to several thousand-fold depending on the fluorophore that is used.^67,68^

Recently, dimer nanoantennas assembled on a DNA origami structure have been used for single-molecule fluorescence bioassays.^69^ In a sandwich assay, the signal from emitters in the hot spots was amplified >400-fold and allowed the detection of DNA fragments with a high signal-to-noise ratio. In a different implementation, Visser et al. proposed the use of a plasmon ruler in different geometries to enable the detection of conformational dynamics of a single biopolymer on time scales of microseconds.^70^

In direct assays, nanosecond single-molecule dynamics have been studied using a combination of label-free plasmon sensing and iSCAT. The motion of nano-objects in the close vicinity of a single gold nanorod was monitored by probing plasmon shifts using iSCAT.^37,71^ In this configuration, a time resolution of 100 ns was achieved, albeit with a complex optical setup. It is evident that existing methods are likely to be inspired by these pioneering studies to access shorter time scales and visualize up to now inaccessible biomolecular phenomena.

In contrast, measuring over long periods of time provides information on the evolution of the concentration of an analyte (Figure 5b). This is highly valuable in diagnostics applications where changes in biomarker concentration are strong indicators of underlying diseases. Biosensors that can continuously monitor analyte concentrations over time are therefore highly sought after but extremely challenging in their development. First of all, the detection system must be insensitive to drift, which can be dominant over long time scales, providing a clear case for single-molecule sensors that exhibit digital signals. In addition the sensor should respond to increasing and decreasing concentrations of the analyte, which can be achieved using low-affinity receptors that generate reversible detection events.

Ensemble-averaged electrochemical sensors capable of continuous monitoring have been reported by the team of Plaxco. In this case, the conformational change of an immobilized aptamer on the surface of an electrode is induced by the binding of the analyte resulting in a variation in the measured current.^72^ The signal generated is dependent on the concentration of the analyte, and the low (micromolar) affinity of the aptamer provides a sensor that is reversible on time scales of minutes. The system has thus been used for the detection of several small molecules such as antibiotics in real time and in live animals.^73^ However, the use of low affinity capture probes here results in a sensor that responds to antibiotic concentrations of micro- to millimolar. The vast majority of biomarkers are present at concentrations of pico- to nanomolar, which are not accessible with this ensemble-averaged approach because the fractional occupancy of the receptors is very low at these low concentrations.

For biosensors with single-molecule resolution, the team of Prins has reported the use of particle mobility sensors for continuous monitoring.^51,54,56,74^ Low affinity receptors are conjugated to a tethered nanoparticle and the sensor substrate in a sandwich or competitive format (see Figure 4). The use of low-affinity receptors again leads to a reversible binding of the particle to the sensor surface. Single-molecule sensitivity now provides the ability to monitor at pico- to nanomolar concentrations because each single binding event can be resolved. This methodology based on affinity binders has shown great versatility in the detection of different analytes (e.g., ssDNA, cortisol, creatinine) with measurements extending over several hours. Increasing and decreasing concentrations can be monitored over time (Figure 4c), validating the use of these sensors for continuous biomolecular monitoring.

For now, the number of available continuous biosensors is very limited but expected to increase in the coming years due to their added value not only in healthcare but also in the monitoring of the environment and food processes. The method critically relies on the availability of low-affinity capture probes: although the affinity of DNA capture probes can be conveniently tuned by their sequence, this is not so straightforward for protein-based capture probes. Nature and bioassays alike often employ antibodies that exhibit affinities of 0.01–10 nM with accompanying (estimated) dissociation rates of 10^–6^ to 10^–3^ s^–1^. It is immediately clear that these interactions are not reversible on time scales of a typical measurement. Further development of antibody mimetics such as affimers, nanobodies, nanofitins, aptamers, or peptides^75^ is therefore crucial to achieving specific but reversible interactions that are needed for affinity-based continuous monitoring.

### Compatibility with Complex Matrices

Most studies and commercial devices are now based on the measurement of samples in buffers or filtered biological fluids. These fluids are very different from the highly concentrated and complex media such as blood, saliva, or urine; chemical reactors; or surface water. The major challenge is 2-fold: first, the complex medium may generate optical signals itself (autofluorescence, scattering by proteins and clusters) that obscure the intrinsically weak single-molecule signals. Sensor designs that achieve selective enhancement of the specific single-molecule signals (based on, e.g., plasmon-enhanced fluorescence or nanoparticle labeling) as described above may be a promising avenue to ensure a high signal-to-noise ratio even in fluids that generate background signals. Second, the unwanted detection of untargeted species often results in a signal even if no analyte is present. To counter this problem, most techniques employ blocking agents (BSA, casein, detergent) or molecules with specific antifouling properties (polymers or peptides).^76^ The development and design of new molecules that can provide an effective antifouling coating are crucial and beneficial as demonstrated by the development of a range of zwitterionic polymers.^76−78^

However, despite the use of optimized antifouling coatings, the generation of nonspecific interactions often remains substantial especially for the detection of low analyte concentrations. The digital nature of single-molecule signals may provide alternative solutions because they provide access to single-molecule kinetic properties (e.g., time between the binding events, and residence time). If the specific interactions exhibit a different kinetic profile than the nonspecific interactions, they can be discriminated by determining their kinetic fingerprint. By statistical analysis of these kinetic properties, the specific and nonspecific interactions can in some cases be distinguished based on their distribution of, e.g., the bound-state lifetime.

**Figure 6:**
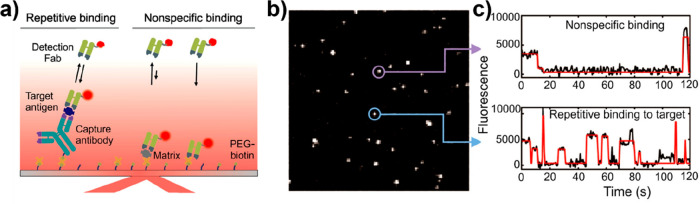
(a) Experimental scheme for the detection of analytes (target antigen) by SiMREPS. (b) Single movie frame of a representative microscope FOV; the bright puncta represent single FPs bound at or near the coverslip surface. (c) Representative intensity versus time traces showing the distinct kinetic fingerprints of nonspecific binding (top) and repetitive binding to the analyte (bottom). Reproduced with permission from ref (39).  Copyright 2020 American Chemical Society.

One implementation of this approach was suggested by the team of Walter who have developed the SiMREPS approach. This method is based on the transient, reversible binding of fluorescent detection probes to immobilized analyte molecules to generate a digital signal that carries the kinetic signature of the analyte–probe interaction. One analyte molecule (captured on the sensor surface) will repeatedly interact with the solution-phase detection probe, resulting in a sequence of single-molecule fluorescence bursts (Figure 6).^39,59^ Nonspecific interactions may also occur on locations where no analyte is captured, but these will mostly not result in the repetitive binding of the detection probe, thus exhibiting a different kinetic profile as can be seen in Figure 6c. This allows for the filtering of nonspecific interactions that improves the specificity and limit-of-detection of the sensor to values that are competitive with ELISA.

Rather than using the bound-state lifetime, it is also possible to use the difference in molecular weight of each molecule in a complex mixture. The magnitude of the optical signal generated by the analyte molecule is directly related to its mass (or volume), resulting in an optical signal or an optical imaging contrast that depends on the molecule’s mass. In this case, no reversible binding of the analyte is needed since the relation between the detector contrast with the molecular weight of the analyte can be performed using a calibration curve recorded in a monodisperse sample. In a polydisperse sample, each protein detection event can then be assigned a unique mass. Such single-molecule mass sensing has been demonstrated using iSCAT,^36^ with plasmonic scattering imaging (PSI)^79,80^ and evanescent scattering imaging (ESI)^81^ to discriminate between signals from different species.

After binding of the analyte to the capture probes, the molecular detection is often done in buffered solutions after a washing step.^54,59^ Molecular detection directly in complex media, without washing steps, has been shown for diluted serum,^39,80^ filtered blood plasma,^56,74,82^ undiluted serum,^49^ and whole blood.^83^ Among these studies, few have succeeded in moving into relevant biological environments. It would appear that despite techniques to discriminate between specific and nonspecific interactions, the noise caused by these interactions is still overwhelming the signal for ultralow concentration. However, it is worth considering that these studies are successful in achieving specific detection in diluted media or even blood serum. It should be noted that the combination of this detection by fingerprinting with the use of blocking or repelling agents is a direction to pursue in order to push the technologies’ compatibility with wash-free single-molecule detection in complex media.

### Multimodal Sensors

**Figure 7:**
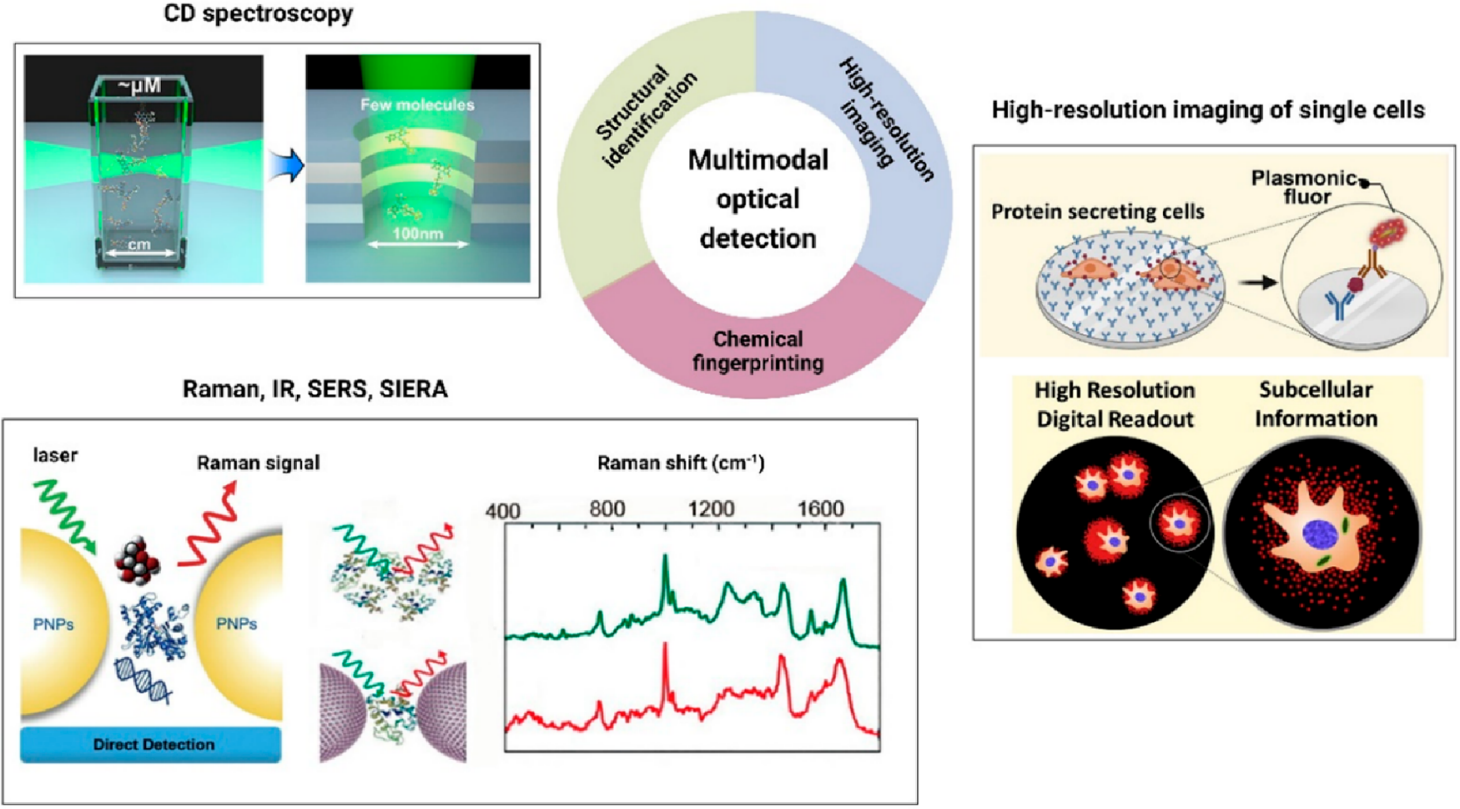
Schematic illustrating the possibilities of multimodal optical detection methods by combining multiple optical techniques for simultaneous structural identification, chemical recognition, and high-resolution imaging of analyte molecules. Reproduced with permission from refs (87 and 91). Copyright 2018, 2022 American Chemical Society. Reprinted with permission from ref (93). Copyright 2022 Elsevier Inc.

Correlative optical microscopy is a new direction that shows great promise in combining complementary optical methods to extract more information from biomolecular processes occurring in complex microenvironments, see Figure 7.^84^ Between early 1980s and mid 1990s, the optics and biophysics community has witnessed the development of a wide gamut of isolated, nondestructive optical tools that were quite capable of providing real-time information on various cellular and molecular phenomena. These include optical tweezers (OTs), multiphoton microscopy, fluorescence lifetime imaging (FLIM) microscopy, nonlinear optical techniques like secondary (SHG) and third harmonic generation (THG) microscopy, coherent anti-Stokes Raman microscopy (CARS), and circular dichroism (CD), among others.^85^ Each of these imaging techniques are unique and yield optical readouts with distinct information on biomolecular processes. However, no single optical technique can be of universal use. Thus, in order to obtain detailed information on complex biological events, it may become essential to design and implement multimodal detection platforms by combining highly specialized optical methods with potential single-molecule resolution. The modality of combining two or more optical methods should be specifically designed and complementary.

On the other hand, Raman and IR spectroscopy have proven to be powerful analytical tools for chemical identification through vibrational fingerprint information.^86−88^ The inherent sensitivity limits of these techniques were further improved by exploiting the concepts of chemical and electromagnetic field enhancement through rational designing of nanoparticle–nanoparticle or nanoparticle–substrate configurations.^89^ In addition to analyte molecule recognition, often useful additional information on reactive intermediates along with the chemical kinetics can be extracted by closely monitoring the single-molecule SERS trajectory.^90^ Although single-molecule SERS has been demonstrated for model compounds, the detection and fingerprinting of single biomolecules remains a tremendous challenge that has not been solved yet.

Circular dichroism (CD) spectroscopy is yet another very widely used optical spectroscopic method based on the differential absorption of left and right circularly polarized light, which renders structural information (conformation) of chiral molecules. As such, since chiral biomolecules (proteins, sugars, nucleic acids, amino acids) are ubiquitous in nature, it will therefore be a relevant technique to apply particularly in the areas of chiro-optical biosensing. Since chiro-optical responses are often weak, the signals could be further boosted by attaching chiral molecules on plasmonic nanostructures, thus enabling a new functionality such as plasmon-enhanced chiro-optical spectroscopy. Recently, Levy’s group has proposed such a plasmon-enhanced chiral sensing method by combining CD with metamaterials.^91^ However, the enhancement achieved by these metasurfaces is limited, and therefore, a complementary plasmonic-enhanced FDCD (fluorescence detected CD) is clearly needed, which at the same time is also capable of observing single molecules.

A particularly powerful approach correlates single-molecule biosensing with spatial mapping, thereby providing spatially resolved single-molecule sensing. Rather than aggregating the molecular detection events to obtain an estimate of the analyte concentration, this approach could be used to, e.g., map single-molecule cell secretion by combining single-molecule sensing with super-resolution localization microscopy.^92^ Understanding the spatiotemporal dynamics of biologically relevant events at a single-cell level is essential to understanding the response of cellular systems to stress and medication. Recently, Seth et al. demonstrated a high-resolution imaging of the protein secretion process at the single-cell level using a plasmon-enhanced FluoroDOT assay, which enables high-resolution spatial mapping of single-molecule secretion.^93^ A more detailed view of single-molecule secretion and its heterogeneity might be obtained by dynamic tracking of single-molecule secretion events that may enable the continuous monitoring of secretion events with nanometric spatial resolution and molecular quantification.^94^

The next generation of correlative single-molecule optical tools is expanding at a promising pace. However, keeping in mind the future directions and applicability of single-molecule optical methods, particularly in emerging areas of high-throughput biosensing, it is imperative to develop a multimodal optical detection platform that is capable of revealing multidimensional information at the single-molecule level. A judicious combination of these previously discussed single-molecule optical detection methodologies could eventually lead to developing next generation multiplexed and multimodal detection platforms with high spatiotemporal and spectral resolution.

### Miniaturization and Integration

The outbreak of the Covid-19 pandemic has acted as a wake-up call for governments and healthcare industries that more attention is needed for the development of technologies for rapid and quantitative diagnostics. With an ever-increasing global demand for precision health and medicine, major advancement in next-generation biosensors is expected to be primarily driven by inexpensive, user-friendly, portable, point-of-care (POC) based sensing devices. Sensor designs based on complete lab-on-a-chip (LOC) detection platforms have been reported already for portable and miniaturized health-monitoring devices.^66,95^ In addition to being portable, these easy-to-use devices offer unique distinct advantages like requiring low sample consumption, providing an ultrasensitive response, ease-of-operation, and on-location health monitoring capabilities. However, quantitative and continuous monitoring of biomolecular markers with high specificity and sensitivity is still lacking in most devices.

Optical single-molecule sensors may provide the ideal solution in a variety of applications; however they still require bulky and expensive optical instruments restricting their use to research laboratories involving trained personnel. With continuous efforts in the field of optical imaging and microscopy combined with parallel growth in microfluidics, it is now possible to detect single molecules based on LOC and POC diagnostic platforms that use enzymatic amplification.^96^

The development of single-molecule affinity-based assays on an integrated and miniaturized platform will be ground-breaking but is yet to be realized. The first step in their development will be the construction of portable devices. Portable microscopes, sometimes equipped with existing hand-held devices such as smartphone cameras, have been introduced by various research groups enabling optical imaging and sensing of single labeled biomolecules.^7,82,97,98^ With most smartphones these days equipped with industrial CMOS sensors, they could be a versatile platform providing possibilities of single molecule sensing using low-cost devices. However, they are still limited in their practicality for biosensing applications as major challenges remain due to a limitation in sensitivity and resolution of cameras and lenses that lack a high numerical aperture.

This limited sensitivity can be partly mitigated by taking advantage of signal-enhancement strategies using, e.g., plasmon enhanced fluorescence. This strategy has recently been employed to push the detection limit of portable microscopes to the single-molecule level.^99,100^ These studies have already shown promising biosensing results showing single-molecule DNA detection, thereby conceiving the possibility toward building more compact and miniaturized single-molecule sensors based on existing hand-held devices. However, the entire process of integration and complete automation of field-deployable, ultrasensitive optical sensors based on existing hand-held portable devices is still in its infancy. Based on current research trends in building miniaturized biosensors, there is still ample scope to further improve the sensitivity and resolution of these devices by implementing new label-free or signal enhancement strategies with multiplexing detection capabilities.^101^

**Figure 8:**
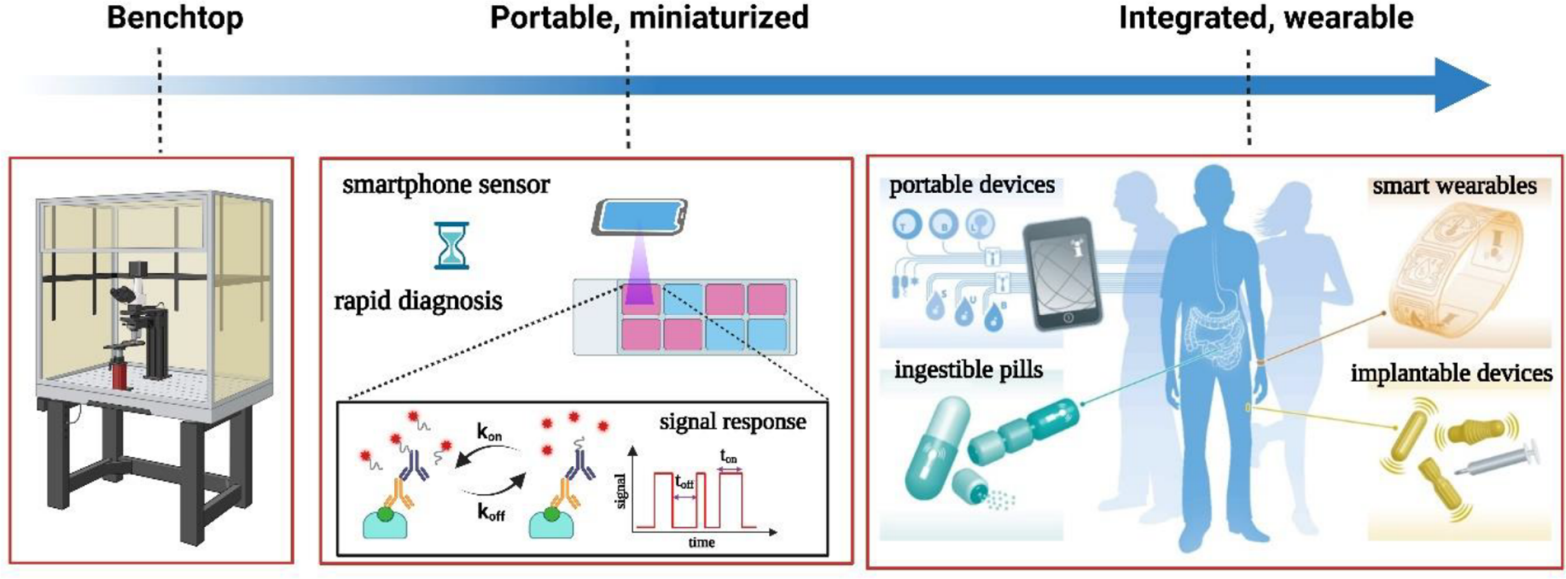
Timeline depicting the evolution of optical biosensors (left, benchtop detection ; middle, rapid, diagnostic field testing kits and portable smartphone based sensors; right, integrated smart biosensors for personalized health monitoring. Reprinted with permission from ref (102). Copyright 2016 Nature Publishing Group.

The ultimate step of miniaturization and integration will likely rely on photonic integrated circuits to achieve millimeter level miniaturization. Future forms of smart diagnosis and personalized health monitoring technologies may come with integrated trackers, sensors, and cameras included in smart pills, smart wearables, and implantable/injectable sensors (Figure 8). These miniaturized integrated devices collect health data by continuously monitoring molecular and physiological parameters.^102^ Photonic integrated circuits may enable this level of integration by combining excitation sources, detectors, and the sensors themselves onto a single semiconductor chip that can be produced in bulk and does not have any moving parts.^76,103^ Advancements in this field heavily rely on synergetic research efforts in combining semiconductor technology (microelectronics, integrated photonics) with optical sensor technology.

It is also worth mentioning that in the current age of artificial intelligence and machine learning technologies, it is sensible to implement smart algorithms which actively take part in decision making and fast diagnosis. This can be followed by a real-time evaluation of the patient’s condition and subsequent personalized treatments. This combination of diagnostics and therapy is often dubbed theranostics and is already implemented in continuous glucose sensors that include an automated insulin delivery system.

## Conclusions

In the past decade, several methods have been developed to achieve the single molecule limit, each with their own benefits and drawbacks. Direct assays are the easiest to implement, however they are limited to analytes of significant size to generate a detectable signal. Sandwich assays can be implemented for bivalent analytes and provide a larger signal by using a tag. However, the tag may experience nonspecific interactions which has prohibited sandwich assays from being performed in undiluted biological matrices without washing. Competitive assays face a similar challenge related to nonspecific interactions of the tags but enable detection of molecules at the expense of assay complexity. Nonspecific interactions are particularly difficult to suppress for large (particle-based) tags, which implies that the usage of small tags is preferred despite their lower optical signals. Although much progress has been made in single-molecule optical sensing, the next phase of development will focus on (1) expanding the functionality of the sensors by expanding the accessible time scales and including multimodal sensing approaches and (2) increasing the practicality of the approaches by compatibilization with complex fluids and miniaturization. The applications of single-molecule optical biosensors is bright and extends beyond the area of healthcare. Most contaminants in surface and sewage water are biomolecular in origin and include painkillers, antibiotics, hormones, and nutrients. The concepts of single-molecule biosensors are therefore equally applicable to environmental monitoring to safeguard water and soil quality. In addition, many food production processes benefit from the monitoring of biologicals that are indicators for taste, freshness, and production efficiency. To conclude, although single-molecule sensors are in their infancy they will cause a paradigm shift in the ability to monitor biomolecular dynamics and concentrations across a broad range of time scales.
